# Supplementary material for: A data-driven characterisation of natural facial expressions when giving good and bad news
Source: PLoS Comput Biol. 2020 Oct 28;16(10):e1008335. doi: 10.1371/journal.pcbi.1008335 (PMC7652307; doi:10.1371/journal.pcbi.1008335)
Supplement: S1 Table — (DOCX) [file pcbi.1008335.s001.docx]

| **S1 Table. List of the ten positive and negative phrases delivered by each subject.** | |
| --- | --- |
| Good news … | I’m sorry to say … |
| … your loan has been approved! | … you haven’t got the job. |
| … you’ve got the job! | … we can’t do anything for you. |
| … the vendor has accepted your offer! | … we’ve sold out. |
| … your tests have come back clear! | … the operation didn’t go well. |
| … your application has been accepted! | … we can’t extend you the loan. |
| … your promotion has been agreed! | … we’ve found some serious structural problems. |
| … we won the match! | … your application was denied. |
| … the operation went well! | … we’re going to have let you go. |
| … your car passed its MOT! | … a break-in has been reported at your house. |
| … your bid has been successful! | … someone has been accessing your account. |
