# Supplementary material for: A data-driven characterisation of natural facial expressions when giving good and bad news
Source: PLoS Comput Biol. 2020 Oct 28;16(10):e1008335. doi: 10.1371/journal.pcbi.1008335 (PMC7652307; doi:10.1371/journal.pcbi.1008335)
Supplement: S2 Table — Significant contrasts are highlighted in bold. (DOCX) [file pcbi.1008335.s002.docx]

| **S2 Table*.* Full post-hoc contrasts of classification accuracies between models.** Significant contrasts are highlighted in bold. | | | | | | | |
| --- | --- | --- | --- | --- | --- | --- | --- |
| Subject | Contrast | *Prefix* clips | | | *Suffix* clips | | |
|  |  | t | df | *p* | t | df | *p* |
| S1 | McGM > AUs | **6.34** | **27** | **< .001** | **2.85** | **27** | **.038** |
|  | McGM > Pose | **22.46** | **27** | **< .001** | **12.87** | **27** | **< .001** |
|  | McGM > AUs+Pose | **4.71** | **27** | **< .001** | 2.02 | 27 | .204 |
|  | AUs > Pose | **16.12** | **27** | **< .001** | **10.02** | **27** | **< .001** |
|  | AUs > AUs+Pose | -1.63 | 27 | .379 | -0.83 | 27 | .839 |
|  | Pose > AUs+Pose | **-17.75** | **27** | **< .001** | **-10.85** | **27** | **< .001** |
| S2 | McGM > AUs | **18.88** | **27** | **< .001** | **12.89** | **27** | **< .001** |
|  | McGM > Pose | **14.84** | **27** | **< .001** | **9.94** | **27** | **< .001** |
|  | McGM > AUs+Pose | **11.51** | **27** | **< .001** | **8.18** | **27** | **< .001** |
|  | AUs > Pose | **-4.04** | **27** | **.002** | **-2.95** | **27** | **.031** |
|  | AUs > AUs+Pose | **-7.37** | **27** | **< .001** | **-4.72** | **27** | **< .001** |
|  | Pose > AUs+Pose | **-3.33** | **27** | **.013** | -1.76 | 27 | .313 |
| S3 | McGM > AUs | **11.49** | **27** | **< .001** | **7.56** | **27** | **< .001** |
|  | McGM > Pose | **9.73** | **27** | **< .001** | **5.17** | **27** | **< .001** |
|  | McGM > AUs+Pose | **6.27** | **27** | **< .001** | **3.38** | **27** | **.011** |
|  | AUs > Pose | -1.76 | 27 | .315 | -2.39 | 27 | .103 |
|  | AUs > AUs+Pose | **-5.21** | **27** | **< .001** | **-4.19** | **27** | **.001** |
|  | Pose > AUs+Pose | **-3.45** | **27** | **.009** | -1.80 | 27 | .297 |
| Cross-subject | McGM > AUs | **5.06** | **87** | **< .001** | **7.52** | **87** | **< .001** |
|  | McGM > Pose | **8.52** | **87** | **< .001** | **8.28** | **87** | **< .001** |
|  | McGM > AUs+Pose | **7.47** | **87** | **< .001** | **7.22** | **87** | **< .001** |
|  | AUs > Pose | **3.46** | **87** | **.005** | 0.76 | 87 | .870 |
|  | AUs > AUs+Pose | 2.41 | 87 | .083 | -0.29 | 87 | .991 |
|  | Pose > AUs+Pose | -1.05 | 87 | .721 | -1.06 | 87 | .716 |
