# Supplementary material for: A data-driven characterisation of natural facial expressions when giving good and bad news
Source: PLoS Comput Biol. 2020 Oct 28;16(10):e1008335. doi: 10.1371/journal.pcbi.1008335 (PMC7652307; doi:10.1371/journal.pcbi.1008335)
Supplement: Attachment — Submitted filename: PCOMPBIOL-D-20-00591_response.docx [file pcbi.1008335.s009.docx]

We thank all the reviewers for their helpful and constructive comments. We have made a number of changes to our manuscript that we feel address the reviewers’ concerns. We provide specific responses to the reviewers’ comments below.

Reviewer #1: The authors develop a multi-channel gradient motion (McGM)model combined with principal components analysis to capture information about facial variance over frames in moving dynamic faces. This representation of the dynamic information was used with a classifier to predict valence very successfully. The authors also show that by “tracing samples along the discriminant dimension and back projecting into the image space” they could visualize and interpret features that give rise to emotional valance. Notably, both internal features as well as head pose/movements were reliable signals of message valence.
To quantify the relationship between the McGM features and valence, the authors
compared these features to the extracted Action Units (AUs), Pose, and AUs + Pose. The results show that the McGM features supported more accurate valence classification than did the
AU and pose models tested. A simple human behavioral test supported the conclusion that human behavior can be tracked by variations in the information extracted by the McGM model. Finally, the authors show some degree of generalization across subject classification for all models, but again, with better generalization for the McGM model.

Evaluation

The work provides a nice illustration of the use of the McGM model for extracting valence information from a dynamic face that is captured during natural movement. On the positive side, the model is elegant and effective, and it makes use of detailed facial motion information in naturalistic stimuli. The manuscript is well-written and the experiments are well-designed to test the utility of the model. On the less positive side, there were some places in the paper that were not entirely clear on methods. Consequently, there are some parts of the procedure that are either problematic or are not well enough described to be sure they do not confound variables in the test. There are also only weak links to theory here (see below). These concerns are described below, along with a number of minor points.

Major Comments.

1. I was unsure about the unit of comparison between the McGM features and the AU and Pose features. From what I understand, the McGM features that were assigned to the category of “positive” or “negative” were based on the motion of the full video sequence (line 154-155), whereas the AU’s were done frame by frame (222-224). In short, I really could not extract from the text, exactly what was done. I would worry about the comparison of a frame-by-frame method to a whole-video motion method comparison. I wonder if this is fair comparison. AUC’s are most accurate in higher-intensity frames. It seems possible that some of the frames then would contain excellent valence information, but the ones in between would contain weak or no valence information. Therefore, classification may be succeeding well on intense frames, but performance measures would be diluted by interim frames.

The McGM here is used simply for the purposes of registration, i.e. registering each frame to a common reference. This yields a 5-channel image (RGB + warps) for each frame which is subsequently vectorised, ultimately yielding 1 sample in the McGM feature space for each and every frame. The number of samples is therefore identical between the McGM and the FACS-based models, the only difference is the dimensionality of the feature spaces (and what those dimensions measure). Note that the two-frame version of the McGM used for image registration here differs slightly from some previous applications of the model in which it was instead used to obtain local velocity estimates at a particular point in time based on extended sequences of frames. We recognise that readers familiar with the model’s previous applications might therefore be confused by its current usage, so we have added extra details to the Introduction (lines 119-122), Results (lines 155-156, 165-166), Discussion (line 341), and Methods (lines 538-541) to clarify this point.

2. The work seems very driven by empirical rather than theoretical concerns, though it is not entirely clear what direction the authors want to take. If this is just a “performance paper”, then more should be said about other computational approaches from face tracking work in the computer literature. If there is a more biological element, some argument should be made on neural or psychological grounds. The finding that behavioral performance relates to McGM features is probably not enough. Note that the Discussion mostly restates the results. Some clarity of the higher level motivation of the work would be beneficial.

The aim of this study was to demonstrate that it is possible to extract a behaviourally meaningful dimension of facial emotion from videos of freely generated facial expressions, and to visualise and quantify the underlying changes in the face that correspond to this dimension. We particularly wanted to establish this method as an alternative to more traditional approaches which often require actors to deliberately pose a pre-selected set of expressions or use a classification approach which was derived from human conception rather than a more objective data-driven procedure.

The procedure is biologically relevant to the extent that it discriminates on the basis of the same input available to human observers, and that the extracted dimension is recognisable to human observers as conveying valance relevant information. We make no particular claims about the specific details of the method as being implemented in human vision, however the fact that it is capable of delivering a meaningful distinction makes it a viable candidate model for human vision.

It is possible other methods may provide better discrimination of the classes. However, efficacy is only one criterion for a biological model: simplicity, biological plausibility, real-time constraints, and empirical testability are more relevant in this case.

Given the importance of these points, we have added some details to the manuscript to help clarify the rationale and better contextualise the model. Firstly, we have updated the Introduction to make more explicit that the aim is to develop a paradigm for extracting facial cues from freely generated behaviours (lines 116-118). Secondly, we have updated the Discussion to acknowledge the availability of other computational models (such as neural networks), but made clear our approach is suitable for our goals and has the advantage of being relatively simple (lines 391-395).

Minor comments:

1. The title seems a bit off the mark for what is actually done in the study.

We have updated the title to more clearly describe the paradigm and key objective (characterising facial expressions when giving good versus bad news).

2. The use of the word “data-driven” should be explained or defined. It seems to make an implicit contrast to AU’s ….but it is not clear.

The technique is “data-driven” in the sense that the dimension of emotional valence is extracted from the data, based on freely generated facial behaviours. This contrasts with more traditional approaches in which actors would be required to deliberately pose specific expressions (e.g. happiness and sadness), which often leads to somewhat artificial caricatures of those expressions. As mentioned in response to Major comment #2, we have updated the Introduction (lines 116-118) to make more explicit the study aim is to use the data to extract facial cues from natural behaviours. We have also updated the study summary in the Discussion (lines 342-343) to reiterate that we provide a data-driven solution to extracting facial cues from natural facial behaviours.

3. Also, “facial expressions” is used a bit loosely here. Valence and expressions are not the same thing. And, one can imagine that expressions (e.g., surprise) can be either positive or negative valence. More care is needed in terminology.

We use the term “valence” here to denote a general dimension of emotional positivity versus negativity, in line with our approach of asking subject to naturally deliver positive or negative messages. The most analogous Ekman-style labels for these expressions would presumably be “happiness” and “sadness”, however we have deliberately avoided using these terms as they might (incorrectly) suggest subjects were encouraged to explicitly express these emotions (e.g. as would be the case in more traditionally posed face stimuli). Nevertheless, we would certainly not wish to suggest that valence must map directly on to happiness / sadness, or that it could not interact with other emotional dimensions (e.g. arousal). We have therefore updated our Discussion (lines 440-443) to acknowledge how valence may be expected to interact with other emotional dimensions.

4. Line 238 finer grained detail – or do you mean motion? Both differ between the elements of the comparison.

The McGM registration process offers fine-grained detail in the sense that it retains pixel-level information about the facial texture and configuration. This contrasts with the FACS-based models that offer coarser scale information about the head position and intensity of the AUs. For instance, whereas AUs could indicate some magnitude of a facial movement has occurred, the McGM could use the pixel-level information to more precisely characterise the subtleties (e.g. changes in texture and shape) of this movement. We have updated the Results (lines 293-294) and Discussion (lines 400-401) to make clearer the importance of this pixel-level detail.

5. Discussion- How much beyond valence detection do the authors think the method will go? Again, the topic of facial expression begins in the intro, but dynamic facial behaviors are the focus of the discussion.

We use the dimension of valence here as a test case, but would suggest that the approach here could be extended to many other dimensions of continuous facial variation. The Discussion (paragraph from lines 430-445) already suggests the technique could be applied to other emotional dimensions such as arousal, and to non-emotional dimensions such as social dominance and trustworthiness. As stated above, in response to Minor Point #3, we have updated the Discussion (lines 440-443) to acknowledge potential interactions between emotional dimensions (e.g. valence and arousal). We have also included (line 445) an example of an additional social dimension (perceived political affiliation), based on a previous study from our lab which identified a potential continuous perceptual dimension of this factor.

Reviewer #2: The current manuscript describes a bottom-up approach for capturing the defining features of positive and negative facial expressions from natural occurring displays. The study involves a novel technique to do this and the authors present their findings in a very comprehensive way. The results reveal that the technique is successful in extracting features of facial displays that are recognized by independent (human) raters as conveying positive and/or negative feelings. The authors discuss how the technique could be extended to other displays.

I found this a very interesting paper that I enjoyed reading. I have a few minor comments and some questions for further clarifications that I will describe below, but I did not have any major reservations about the methodology (as far as I understood it) and the interpretation of the findings.

Before giving my specific comments I have to declare that I have insufficient knowledge of Multi-channel Gradient Modelling, the employed method of Discriminant Analysis and Hyperalignment to judge whether these techniques were appropriate and executed correctly. However, I should say that I found the descriptions and explanations of these techniques very clear. In my review I focus on some aspects of these techniques that were less clear to me and that could perhaps benefit from some further explanation, and aspects of the work related to the FACS codes of the extracted images and the ratings involving human observers.

First, I think some more can be said about the FACS codes of the extracted displays. Interestingly, there appears to be very little overlap in the patterns of FACS codes extracted from the different subjects (Figures S1 and S2). The fact that the human raters were able to differentiate the back-projected images in terms of their valence consistently between subjects, despite the fact that there was such disparity between subjects in terms of the morphological features of the displays is something that could be discussed further. The most consistent pattern of Action Units (AUs) associated with positivity/negativity across subjects were AU1, AU12, and AU17. The relevance of these AUs in this context could be discussed further. Finally, with regards to head movement there is in fact literature that suggests that an upward tilt is associated with positive feelings, while a downward tilt is associated with feelings of dejection (Haidt & Keltner, 1999; Tracy & Robins, 2004; Tracy, Robins & Schriber, 2009) and this could be discussed as well.

This is an important point. We have therefore expanded our discussion of the role of FACS metrics in the facial behaviour exhibited here. In the Results (lines 235-245) we have provided a more detailed description of the pattern of FACs codes from images projected along the discriminant dimension, including identifying both commonalities and idiosyncrasies over subjects. We have also moved the figures displaying these measures from the supporting information into the main text (now Figs 5-6). Finally, we have also included a discussion of the FACs codes of the projected images and their relationship to the behavioural ratings of these images in the Discussion (lines 418-429).

With regards to the previous literature on head pose, we have included references to these studies along with a discussion of their relevance to expressions of embarrassment / shame and pride (lines 370-374). We thank the reviewer for bringing this literature to our attention.

Somewhat related to this, the cross-subject decoding accuracy is lower than the decoding accuracy for the individual subjects. This relates to the generalizability of the findings and is something that could be elaborated on more thoroughly in the discussion section. The absolute levels of ratings by the observers of the different subjects are also relevant in this context. For example the displays of S1 seem to be more clearly differentiated by raters than the displays of the other subjects. What may be the reason for this?

This is a good point, and we have now included a greater discussion of the presence of individual differences between subjects. In particular, as stated above, we now include a discussion (lines 418-429) of the behavioural ratings alongside the FACs codes for the projected images.

The reasons for the greater magnitude of valence ratings for Subject 1 are somewhat unclear. Subjectively, she did seem to show a greater range of expression in her facial behaviours, as can be seen in the more pronounced expressions observed in the back-projected images (Fig 4). However, the LDA decoding accuracies from the McGM-PCA model (Fig 3) do not seem appreciably higher for Subject 1 than the other subjects, although this may be limited by a ceiling effect. Decoding accuracies based on the AUs-only and AUs+Pose models are somewhat higher than for other subjects, but the Pose-only model appears comparable or poorer. Furthermore, the FACs codes extracted for the projected images (Figs 5-6) do not show an obviously greater degree of modulation for Subject 1 over the subjects. Thus, while we can subjectively see a greater expressive range for this subject, our current statistical measures do not seem to capture this. Instead, we have simply noted that behavioural ratings for her indicate a greater modulation of valence, and drawn attention to the subjective appearance of the projected images displayed in Fig 4 (lines 418-422).

One potential limitation of the current technique is that, while the extracted displays are based on filmed displays that involve movement, the back-projected images themselves are static. To what extent is movement an important feature for the accurate decoding of the images? Somewhat related to this, to what extent do the raters rely on making an internal comparison between the images that have been presented to them to judge their positivity / negativity?

The motion information present in dynamic displays surely does carry important information, though static cues appear sufficient to discriminate the emotions. For instance, the FACS-based models do not incorporate motion, but do nevertheless discriminate the emotion above chance. Furthermore, the behavioural ratings of the back-projected images do vary reliably despite the images being static. The temporal dynamics of motion patterns in dynamic facial behaviours is a focus of research currently being conducted in our lab, but as yet is beyond the scope of the current paper.

With regards to raters forming an internal comparison of the images, we would firstly point out that the images were presented in a randomised order, which would make the range of available images less apparent to the raters. Secondly, and more importantly, we feel that even if raters did develop a familiarity with the range of images, this still wouldn’t affect the conclusions of the experiment. Raters were not provided with feedback on their responses, and thus would have no way of calibrating their responses to be in line with our expectations. Consequently, if raters simply could not perceive the valence of the images, then their responses would have appeared uncorrelated with the discriminant dimension. While an internal comparison of the images might have assisted raters in calibrating their response scale (e.g. developing a perception of the extremes or mid‑point of the scale), they would still have to be able to reliably perceive the valence in order to produce the responses that do correlate with the discriminant dimension. To clarify this we have updated the Methods to explicitly state that the trials were presented in a randomised order (line 671) and that no feedback was provided to the raters (lines 675-677).

Some minor comments and questions:
- There seems to be a large overlap between the displays extracted from the prefix and the suffix parts of the sentences. Can the extend of this overlap be tested more formally?

Testing this relationship formally would present a number of challenges. Comparing the back‑projected images directly (e.g. by pixel correlation) would be uninformative as the images would be expected to show very high correlations anyway (e.g. due to common background / foreground areas in the image). An alternative would be to compare the scores within the PCA spaces; however, this is complicated by the fact that separate PCAs are conducted on the *prefix* and *suffix* clips and thus produce two separate PCA spaces. For instance, different components may emerge between the spaces, and even common components may emerge in different orders and / or with different signs. Thus, the PCA spaces are likely to correspond poorly even if representations are relatively similar. A formal test of the similarity then would be quite involved and would require introducing a number of additional methods to allow direct comparisons between the representational spaces. We have, however, updated the Discussion (lines 351-353) to draw attention to the visual similarity evident in the back-projected displays. While this is ultimately subjective, it is more readily interpretable than the more formal alternatives would be.

- Table 2 reports the comparison between the McGM-PCA model and the FACS based models based on planned contrasts, but this is not sufficiently justified. Also, there is potentially relevant information in the comparison between the different FACS based models. Can the post hoc comparisons be reported instead?

The aim of these tests was to compare the performance of the McGM-PCA model against the FACS-based alternatives, and thus a series of planned contrasts against the McGM-PCA seemed the most statistically justifiable. The reduced number of multiple comparisons compared to the full post-hocs offers greater statistical power, and the smaller number of tests are more readily interpretable.

Nevertheless, we appreciate that readers may also be interested in comparisons between the FACS models, and so have included the full set of comparisons in the supporting information (S2 Table). Importantly, despite the reduced statistical power, all of the relationships from the planned comparisons still hold (the McGM-PCA model outperforms all of the FACS models in all but one of the comparisons). Between the FACS models themselves, for the within‑subjects analyses, the AUs+Pose model tends to outperform the AUs- and Pose-only models, while the relationships between the AUs- and Pose-only models are more variable over subjects. For the cross‑subject analyses, the AUs-only model outperforms the Pose-only model for *prefix* clips only, while no other comparisons between the FACS models reached significance. A brief overview of these comparisons is also provided in the Results (lines 288-291, 327-330).

- It wasn’t clear to me how the classification accuracies for the cross-subject FACS codes were calculated. Can some more information about this be provided?

Classification proceeds in much the same manner as for the within-subject analyses. The LDA is trained on the inner-training set (2/3 subjects for one of the two outer-testing phrases), and is then tested on the inner-testing set (1/3 subjects for the other outer-testing phrase). The trained LDA algorithm is used to generate a set of predicted class labels for each sample within the inner-testing set, which are then compared to the true labels to assess classification accuracy. This is repeated for all 6 folds of the inner cross-validation, and then all over again for the 5 folds of the outer cross-validation. This will ultimately yield one set of classification accuracies for each of the 6 × 5 = 30 cross-validation folds, which are then averaged over the folds to produce the accuracies shown in
Fig 3 and the confusion matrices in Fig 8. We have expanded the relevant details in the Methods (lines 617-622) to clarify this.

- This may be a naïve question, but is it not relevant to also look at the variability of the LDA classifications when reporting the classification accuracy? Is there a Standard Deviation or Standard Error that is associated with the Mean proportion of LDA classifications? If so, can this be reported in Figure 2?

We have updated the confusion matrix figures (Figs 2 and 8) to also label the standard deviations of the classifications in the text annotations. For the within-subject analyses (Fig 2) this represents the standard deviations over the 3 subjects, and for the cross-subject analyses (Fig 8) this represents the standard deviations over the 30 cross-validation folds.

- Stratou, Van der Schalk, Hoegen & and Gratch (2017) employed a PCA based methodology on automatically extracted FACS codes of natural occurring expressions to uncover reliable patterns of facial activity that correspond to certain behaviors. It may be relevant to look into this work.

We have now included a reference to this study and a brief discussion of its relevance to our work (lines 403, 406-407).

References

Haidt, J., & Keltner, D. (1999). Culture and facial expression: Open-ended methods find more expressions and a gradient of recognition. Cognition and Emotion, 13, 225–266.

Stratou, G., Van Der Schalk, J., Hoegen, R., & Gratch, J. (2017, October). Refactoring facial expressions: An automatic analysis of natural occurring facial expressions in iterative social dilemma. In 2017 Seventh international conference on affective computing and intelligent interaction (ACII) (pp. 427-433). IEEE.

Tracy, J. L., & Robins, R. W. (2004). Show your pride: Evidence for a discrete emotion expression. Psychological Science, 15, 194–197.

Tracy, J. L., Robins, R. W., & Schriber, R. A. (2009). Development of a FACS-verified set of basic and self-conscious emotion expressions. Emotion, 9, 554–559.

Reviewer #3: This is a nice study, showing how a relatively simple model can extract valence data from naturalistic videos. Using such videos is an important: for some years the expression literature had got bogged down in a self-perpetuating circle of 6 basic expressions and their defining AUs. Another positive feature is that the model is invertible, allowing reconstruction of prototypical stimuli for positive and negative valence.

The least convincing aspect of the work is the use of multiple samples from each of only three participants. It is less surprising that a model can extract commonalities within a relatively self-similar set of videos - especially for the prefix sections. It is therefore reassuring that the model produces almost identical results for the much more varied suffix sections. There is also some testing between participants, which produces results well above chance.

I had a number of queries as I read through the first part of the paper, which were answered when I got to methods. Combined with what looks like a full set of code on the osf site, I think the methodology is well-described.

I think it might be helpful to reference previous work using PCA and LDA to classify naturalistic face images for identity: e.g. Dahl CD, Rasch MJ, Bülthoff I, Chen C-C. Integration or separation in the processing of facial properties - a computational view; Kramer RS, Young AW, Day MG, Burton AM. Robust social categorization emerges from learning the identities of very few faces

We thank the reviewer for bringing this literature to our attention. We have included references to these studies and a brief discussion of their implications in our Discussion sections (lines 386-390).

L165 'then tested on all samples for the held out phrase.' Am I understanding correctly, this should say phrases, one positive and one negative? Again at L484

The plurality of the term is a little ambiguous. One phrase *from each class* is held out at a time, however there are two classes so ultimately two phrases are held out overall. We have tried to clarify this by updating the manuscript throughout to retain the singular “phrase” but to also note that this is per each class. For example: “… a test set comprising all samples in all clips for the remaining phrase *in each class*” (Methods, line 583).

Fig 4 is quite convincing, but I expect animations would be better: could you put some in supplementaries, or your OSF site?

This is a good suggestion. We have included 6 additional video files in the supporting information (S1-S6 Video) showing animations of the projections.

L202 Doesn't seem quite right to put these in supplementaries and then talk about them in main text. Lack of space? Note to editor, the first one needs to be in the main text - second can go to S. I think it would be worth drawing attention especially to the yaw data (and explain that is a turn of the head) since two of the subjects show strong correlations, in the opposite direction. Illustrates why within subject model works much better than between. I'd be more inclined to put table 1 and 2 in supplementaries; the numbers don't add much.

We have moved the supplementary figures into the main text – they now comprise Figs 5 & 6.

L498. The iterative, rolling average description confused me, given that there are only three Ps here. I thought it might go round a loop. The description that follows is simpler - the rolling average bit is a more general description for any N. Because we know N=3, it serves to confuse, here. You could say, align the second to the first, then align the third to the average of the first two in a single sentence.

Yes, the phrasing was intended to describe the general-purpose method that could be applied to any number of subjects. However, we can see that this description might appear confusing with only 3 subjects, so we have updated the methods in line with your suggestion (lines 597-599).

L540 time? Line, I think.

Thank you - we have corrected this typo.

Peter Hancock
